# Prevalence and associated factors of acute diarrhea among under-five children living in Hargeisa Internally Displaced Persons, Somaliland: a community-based cross-sectional study

**DOI:** 10.11604/pamj.2024.47.10.35958

**Published:** 2024-01-10

**Authors:** Mohamed Ahmed Ismail, Mohamed Mussa Abdilahi, Barkhad Aden Abdeeq, Mohamed Jama

**Affiliations:** 1 College of Medicine and Health Sciences, Department of Public Health, University of Hargeisa, Hargeisa, Somaliland,; 2 College of Medicine and Health Sciences, Department of Nursing, University of Hargeisa, Hargeisa, Somaliland,; 3 Department of Child Survival, Save the Children International, Hargeisa, Somaliland,; 4 College of Applied and Natural Science, Faculty of Statistics and Data Science, University of Hargeisa, Hargeisa, Somaliland

**Keywords:** Childhood diarrhea, associated factors, community-based cross-sectional study, Hargeisa, Somaliland

## Abstract

**Introduction:**

in developing countries, diarrhea is a major cause of child death among those under five years old. Dehydration, malnutrition, delayed physical development and early childhood mortality are the major consequences of diarrheal diseases. In Somaliland, diarrheal diseases have been endemic and a major problem since 1994, with epidemics occurring annually. This study aimed to assess the prevalence and risk factors of acute diarrhea among children under five years old living in Hargeisa Internally Displaced Persons (IDPs), Somaliland.

**Methods:**

a community-based cross-sectional study was conducted among mothers of children under five from August to September 2020 in Hargeisa IDPs. A total of 383 mothers were selected using single population proportional formula. Data was entered, cleaned, and analyzed using SPSS version 22. To explore the association between variables, bivariate logistic regression was performed for each independent variable with the dependent variable. Variables with a p-value of < 0.05 were adjusted in multivariate logistic regression. Finally, variables with a p-value < 0.05 were recognized as determinants of acute diarrheal disease.

**Results:**

the prevalence of diarrhea among children under five living in Hargeisa IDPs was 51% (95% CI: 46%-56%). Children older than one year (AOR= 3.59, 95% CI: 2.05-5.20), those not exclusively breastfed (AOR= 4.01, 95% CI: 3.27-4.60), those not given colostrum milk (AOR= 36.41, 95% CI: 25.76-47.90), those drinking water stored in jerry-cans (AOR = 4.90, 95% CI: 1.31-8.39), and those with poor hand washing practices (AOR = 5.74, 95% CI: 1.38-7.82) were more likely to develop diarrhea than their counterparts.

**Conclusion:**

this study concludes that the prevalence of diarrhea was very high (51%). Lack of awareness of exclusive breastfeeding and colostrum feeding, storing drinking water in unprotected containers, and poor hand-washing practices were identified as significant predictors for childhood diarrhea (p-value < 0.05).

## Introduction

According to the WHO, diarrhea is defined as “the passage of three or more loose or liquid stools per day” [1]. It is usually a symptom of an infection in the intestinal tract, which has multiple etiologic agents, including bacteria, viruses, and parasites [2-4]. Diarrheal disease is the second leading cause of death in children under five years old globally. It is estimated that there are 2.5 billion cases and 1.5 million deaths annually in children under five years [5,6]. In developing countries, diarrhea is the major cause of child death when children are less than five years old [6].

The morbidity and mortality associated with diarrhea are multifaceted, stemming from a complex interplay of various factors that significantly impact the health and development of children. In contexts marked by poor socio-economic status and inadequate access to safe water, families often contend with compromised hygiene and sanitation, heightening the risk of diarrheal diseases [7]. The absence of proper handwashing practices, poor housing conditions, and unsanitary waste disposal further compound this vulnerability, contributing to the pervasive nature of diarrheal illnesses [7]. Moreover, improper feeding practices exacerbate the severity of consequences, perpetuating malnutrition and prolonged recovery times [7]. The consequences of diarrheal diseases extend beyond immediate health impacts to include delayed physical development and early childhood mortality [8]. Dehydration, a common outcome of severe diarrhea, poses a serious threat to the body's physiological balance, emphasizing the need for prompt and effective interventions [8]. Additionally, the persistent loss of nutrients during diarrheal episodes, coupled with associated malnutrition, hampers the natural progression of physical milestones [8]. This delayed development not only compromises the child's current health but also sets the stage for potential long-term consequences. Understanding these intricate relationships is crucial for informed interventions to alleviate the burden of diarrhea on the health and development of children.

In Somaliland, where diarrheal diseases have been endemic and a major concern since 1994, with annual epidemics, the health landscape is significantly shaped by very poor sanitation conditions and the widespread consumption of unsafe water by the population. These contributory factors are particularly pronounced in IDPs camps, where living conditions are even more challenging, serving as focal points for endemic cholera outbreaks [9]. The inadequate sanitation infrastructure within these IDP camps exacerbates the risk of waterborne diseases, creating a fertile ground for the transmission of diarrheal pathogens. The inhabitants, often facing displacement due to conflict or environmental factors, contend with compromised hygiene facilities, limited access to clean water, and overcrowded living conditions, all of which amplify the vulnerability to diarrheal infections.

In Somalia and Somaliland, the divergence in prevalence rates, with Somalia at 11% and Somaliland at 47%, underscores the need for a closer examination of the contextual factors influencing these disparities [10,11]. Notably, both studies identified common risk factors contributing to the elevated prevalence of acute diarrhea, including a lack of maternal knowledge regarding handwashing at critical times, the consumption of unsafe water, and the failure of exclusive breastfeeding. Extending the analysis to neighboring countries enriches the comparative framework. Ethiopia, for instance, exhibited a broader range of prevalence rates, spanning from 14.5% to 36.5%, highlighting the heterogeneity within the region [12-15]. Conversely, Kenya reported lower prevalence rates, ranging from 10.6% to 19.6%, when compared to Ethiopia [16]. This variation suggests that while some commonalities may exist in risk factors, there are also distinct contextual elements contributing to the prevalence of acute diarrhea across East Africa.

Understanding the nuanced context of diarrheal diseases in Somaliland is imperative. Delving into the prevalence rates, risk factors, prevention strategies, and treatment options will provide a comprehensive foundation for this study. Annual incidence rates, demographic variations, and the specific challenges faced by populations in IDP camps will be examined to inform targeted interventions and public health policies. Additionally, an exploration of socio-economic determinants, environmental conditions, and healthcare access will contribute to tailored prevention strategies and improved healthcare delivery for this prevalent health concern in Somaliland. Therefore, this study aimed to determine the prevalence of acute diarrhea and associated factors among under-five children living in Hargeisa IDPs, Somaliland.

## Methods

**Study settings:** a community-based cross-sectional study was conducted among mothers of children under five from August to September 2020 in IDP settlements. Hargeisa, the capital city of Somaliland, is situated in the northern part of Somalia at latitude 9°.5624" and longitude 44°.177", with an elevation of 1,334 meters (4,377 feet) above sea level. The city has a population of approximately 1.5 million individuals and features one referral public hospital, eight private hospitals, twenty-five private clinics, and ten public Maternal and Child Health centers (MCHs). Within Hargeisa, there are 16 IDP settlements, comprising 11,549 households with a total population of 71,606 individuals. Among these, 46,544 (65%) are women, and 17,902 (24%) are children under the age of five. The study focused on this dynamic urban environment to investigate the prevalence and associated factors of diarrhea among under-five children in the IDP settlements.

**Study population:** the focus of this study is on mothers who are primary caregivers to children under the age of five, residing within the Hargeisa IDPs community in Somaliland. To be eligible for participation in this study, mothers must have at least one child under the age of five. Additionally, they should be current residents of the Hargeisa IDPs and express a willingness to engage in the research process. By including mothers with children in this specific age group, the study aims to capture data relevant to the prevalence and associated factors of acute diarrhea in the under-five population. Conversely, individuals who did not meet the inclusion criteria, such as mothers without children under the age of five or those residing outside the Hargeisa IDP community, were excluded from participation. Unwillingness to take part in the study is also considered an exclusion criterion. These criteria serve to refine the study's target population, ensuring a focused and relevant investigation into the prevalence and associated factors of acute diarrhea among under-five children in the Hargeisa IDP community.

**Sample size determination:** the sample size was calculated using a single population proportional formula:


$$n=\frac{z^{2}*p*(1-p)}{d^{2}}$$

With the following parameters: n is the sample size; z is the confidence interval at 95% which is 1.96; p is the expected proportion of children with diarrhea, set at 47% (0.47) based on a study in the Sanag region [11]. d is the margin of error (0.05). After substituting these values into the formula, the calculated sample size was determined to be 383 mothers/caregivers.

**Figure 1:**
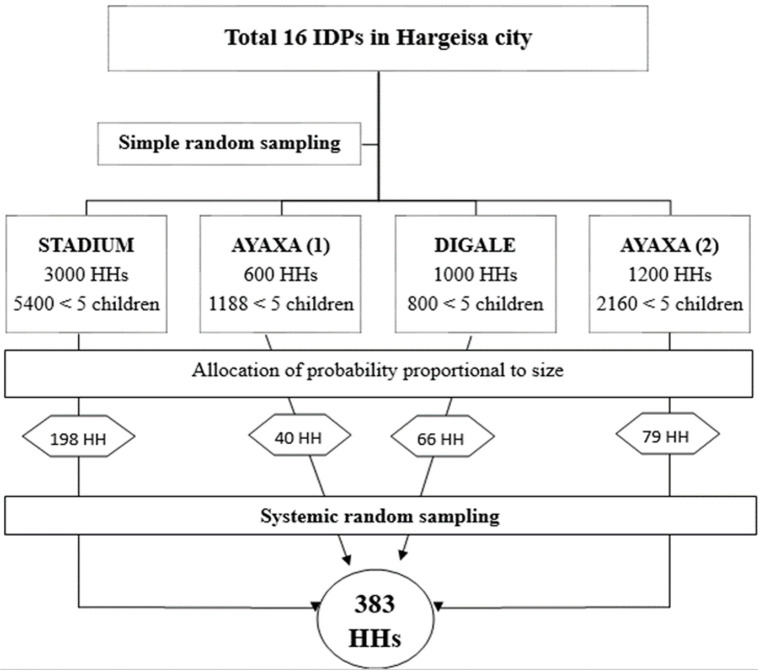
sampling procedure of the study participants

**Sampling procedure:** the study employed a two-stage cluster random sampling technique. The overall sampling procedure is illustrated in Figure 1. First, the total population of IDPs was divided into clusters (stage one) based on their location. Four IDP camps, namely Stadium, Ayaxa 1, Digale, and Ayaxa 2, were then randomly selected from the total of 16 IDPs in the city, utilizing a simple random sampling method, specifically the lottery method. In the second stage, each selected IDP was allocated a probability sample proportional to its size to ensure a representative sample. To identify households with under-five children, registration books maintained by health extension workers in MCHs were consulted, containing information on the number of households with young children in each IDP. For the actual selection of households, a systematic random sampling approach was employed. This involved establishing a sampling interval based on the total number of households in each selected IDP. Starting from a randomly selected point, households were systematically sampled at regular intervals until the desired sample size was achieved. In households where there were two or more children under the age of five, a systematic method was employed to select the youngest child for inclusion in the study. This approach ensured a fair representation of the target population, and the chosen child became the focus of our investigation.

**Study variables:** the dependent (outcome) variable of this study was the status of acute diarrheal disease in under-five children as reported by the mother/caregiver of the child two weeks before the survey. Additionally, to enhance the reliability of the information gathered, the study sought confirmation from health records available at the MCHs in the study area. The independent variables were socio-demographic characteristics of the mother (maternal age, occupation, educational level, household income, and household size), child characteristics (child´s age, child´s sex, vaccination status, exclusive breastfeeding practice, and colostrum feeding), environmental and hygienic factors (water storage container, hand washing practice at critical times, household floor type and frequency of latrine utilization).

### Operational definitions

**Diarrhea:** defined as the passage of three or more loose or liquid stools per day according to WHO criteria.

**Under five children:** refers to children aged less than five years old.

**Prevalence of acute diarrhea:** the percentage of under-five children in Hargeisa IDPs experiencing three or more loose or liquid stools per day during the study period.

**Exclusive breastfeeding:** the practice of feeding an infant only breast milk, without any additional food or drink.

**Colostrum milk:** the first milk produced after giving birth, rich in nutrients and antibodies, given to the newborn in the first few days of life.

**Hargeisa IDPs:** internally displaced persons residing in Hargeisa, Somaliland.

**Determinants of acute diarrheal disease:** variables significantly associated with the likelihood of under-five children developing acute diarrhea based on statistical analysis.

**Data collection procedure:** a structured questionnaire was initially adopted by reviewing existing literature and was first prepared in English and subsequently translated into Somali. To ensure consistency and stability test-retest reliability was conducted and validity was assessed by having subject matter experts. Then, a questionnaire was tested by 10% of the sample size. The finalized questionnaire was then employed for face-to-face interviews with study participants. During the data collection process, health extension workers, who played a crucial role in the IDP set-up, were engaged. These workers maintained registration books in MCHs, serving as valuable references for identifying households with under-five children. Trained health professionals administered the structured and pretested questionnaire, ensuring the accuracy and reliability of the data collected. This approach not only facilitated the data collection process but also leveraged the existing infrastructure and local resources within the IDP set-up.

**Data quality control:** continuous guidance and supervision from the principal investigator were provided to maintain data quality. The data collectors underwent a one-day training session covering the study instrument, consent form, interviewing techniques, and data collection procedures. Data underwent thorough checks for reliability, and a pretest of the questionnaire was conducted with 10% of the sample size.

**Data processing and analysis:** the completeness of the data was visually checked and coded using a whiteboard marker. It was then entered, cleaned, and analyzed using SPSS version 22. Frequency tables were employed to summarize the socio-demographic characteristics of the study participants and the magnitude of diarrhea. To explore the association between the status of diarrhea and risk factors, bivariate logistic regression was conducted for each independent variable with the dependent variable. Variables with a p-value of < 0.05 were then adjusted in multivariate logistic regression. Finally, variables with a p-value < 0.05 were identified as determinants of acute diarrheal disease.

**Ethical considerations:** the study protocol was reviewed and approved by the ethical review committee of University of Hargeisa (Ethical approval No. DRCS/67/05/2021). Prior to data collection, explicit written or oral consent was obtained from the respondents, including parents and caregivers. Informed consent was specifically acquired from all parents and/or legal guardians for participants under 18 and illiterates. Permission to conduct this study was secured from relevant authorities in Hargeisa city. Data collection was carried out with strict confidentiality, adhering to all relevant institutional guidelines and regulations.

## Results

**Socio-demographic characteristics of mothers and their children:** a total of 348 mothers/caregivers participated in this study, resulting in a response rate of 90.8%. The majority of mothers 158 (45.4%) were aged between 26 and 35 years old. One hundred and fifty (150) (43.1%) of them were illiterate, whereas only 30 (8.6%) had a diploma level of education or above. In terms of occupation, more than two-thirds (233 or 67%) of mothers were housewives. Additionally, more than half of the households (187 or 53.7%) had 5 to 8 individuals, and only 58 (16.7%) had more than eight individuals. Furthermore, fifty-five percent of households had 2 to 4 under-five children, and half of them reported a monthly income ranging between 50 USD and 100 USD (Table 1).

**Table 1:** socio-demographic characteristics of mothers and their children living in Hargeisa internally displaced persons Somaliland 2020

Variables	Categories	N	%
**Maternal age (348)**	15 - 25	63	18.1
	26 - 35	158	45.4
	36 - 45	86	24.7
	> 45	41	11.8
**Maternal education (348)**	Illiterate	150	43.1
	Primary level	133	38.2
	Secondary level	35	10.1
	Diploma level and above	30	8.6
**Maternal occupation (348)**	Housewife	233	67.0
	Merchant	72	20.7
	Government employer	12	3.4
	Salaried woman	31	8.9
**Household size**	2 - 4	103	29.6
	5 - 8	187	53.7
	> 8	58	16.7
**Number of under-five children**	Only one	152	43.7
	2 - 4	190	54.6
	> 4	6	1.7
**Household monthly income (USD)**	50$ - 100$	180	51.7
	101$ - 150$	73	21.0
	151$ - 200$	39	11.2
	> 200$	56	16.1

**Demographic and health characteristics of targeted children:** more than half (52.6%) of the children were male. Of them, 23.3%, 37.9%, and 38.8% were aged less than one year, 1-2 years, and 3-5 years, respectively. Two hundred and seven (59.5%) of the children received measles vaccination. About one-third (31.6%) did not feed exclusively on breast milk, whereas eighty percent of them received colostrum (Table 2). One hundred seventy-eight (51.1%) children had diarrhea two weeks before data collection, with the majority of cases (82%) being of the watery type.

**Table 2:** demographic and health characteristics of indexed children living in Hargeisa Internally Displaced Persons Somaliland 2020

Variables	Categories	N	%
**Child’s sex (348)**	Male	183	52.6
	Female	165	47.4
**Child’s age in years (348)**	< 1	81	23.3
	1 - 2	132	37.9
	3 - 5	135	38.8
**Measles vaccination**	Yes	207	59.5
	No	141	40.5
**Exclusive breastfeeding (EBF) practice**	Yes	238	68.4
	No	110	31.6
**Colostrum feeding**	Yes	278	79.9
	No	70	20.1

**Environmental and hygienic characteristics:** majority of households (274 or 78.7%) used a metal tank for water storage. One hundred (28.7%) of children rarely practiced hand washing at critical times, whereas 40.2% and 31% of them sometimes and often practiced handwashing, respectively. In terms of livestock ownership, forty-three percent of households had at least one kind of livestock, mainly goats. Only 58 (16.7%) of households had a mud floor. All households had toilets; however, more than half (51.7%) of children rarely used those latrines (Table 3).

**Table 3:** environmental and hygienic characteristics of households in Hargeisa Internally Displaced Persons Somaliland 2020

Variables	Categories	N	%
**Water storage container**	Jerry can	74	21.3
	Tank	274	78.7
**Hand washing practice**	Rarely	100	28.7
	Sometimes	140	40.2
	Often	108	31.0
**Livestock ownership**	Yes	150	43.1
	No	198	56.9
**Household floor**	Mud	58	16.7
	Cement	217	62.4
	Tiles	73	21.0
**Latrine utilization**	Rarely	163	51.7
	Sometimes	78	24.8
	Often	74	23.5
**Waste management**	Poor	253	72.7
	Good	95	27.3

**Prevalence of diarrhea among under-five children:** the study assessed the prevalence of diarrhea among children under the age of five in Hargeisa IDPs. The two-week prevalence was found to be 51% (95% CI: 46%-56%). This high prevalence indicates a substantial burden of acute diarrhea in the studied population during the specified time frame.

**Factors associated with diarrhea:** in bivariate regression analyses, significance variables (p-value ≤ 0.05) contributing to the occurrence of childhood diarrhea included maternal occupation, household monthly income, child´s age, measles vaccination, exclusive breastfeeding practice, colostrum feeding, water storage container, hand washing practice, livestock ownership, floor materials of the household, frequency of latrine utilization and waste management (Table 4). The significant variables (p-value ≤ 0.05) during bivariate analysis were further considered in multivariate regression analysis to control potential confounders (Table 5). Accordingly, being a child age older than one year; having poor hand washing practices; being from mothers who did not breastfeed their children exclusively, being a child who did not receive colostrum milk, and storing drinking water in jerry-cans were found to be independent predictors of diarrheal disease in children.

**Table 4:** binary logistic regression analysis of factors associated with diarrheal diseases among under-five children living in Hargeisa Internally Displaced Persons Somaliland

Variable	Categories	Diarrhea occurrence		COR (95% CI)	P-value
		No	Yes		
Maternal occupation	Housewife	138	95	1	
	Merchant	12	60	7.263 (3.707, 14.231)	0.000*
	Salaried woman	11	20	2.641 (1.210, 5.766)	0.015*
Household monthly income	50$ -100$	94	86	1.931 (1.026, 3.636)	0.041*
	101$ - 150$	29	44	3.203 (1.542, 6.653)	0.002*
	151$ - 200$	9	30	7.037 (2.770, 17.878)	0.000*
	> 200$	38	18	1	
Child’s age (years)	< 1	51	30	1	
	1 - 2	62	70	1.919 (1.090, 3.380)	0.024*
	3 - 5	57	78	2.326 (1.321, 4.096)	0.003*
Measles vaccination status	Yes	120	87	1	
	No	50	91	2.510 (1.614, 3.905)	0.000*
Exclusive breastfeeding practice	Yes	137	101	1	
	No	33	77	3.165 (1.954, 5.125)	0.000*
Colostrum feeding	Yes	167	111	1	
	No	13	67	33.601 (24.312, 40.489)	0.000*
Water storage container	Jerry can	18	66	11.933 (5.513, 25.831)	0.000*
	Tank	162	112	1	
Hand washing practice of child	Rarely	36	64	3.869 (2.175, 6.882)	0.000*
	Sometimes	60	80	2.902 1.715, 4.912)	0.000*
	Often	74	34	1	
Livestock ownership	Yes	52	98	2.780 (1.790, 4.316)	0.000*
	No	118	80	1	
Floor materials of household	Mud	21	37	3.597 (1.743, 7.425)	0.001*
	Cement	100	117	2.389 (1.369, 4.167)	0.002*
	Tile	49	24	1	
Frequency of latrine utilization	Rarely	54	109	5.094 (2.792, 9.296)	0.000*
	Sometimes	48	30	1.577 (0.798, 3.116)	0.189
	Often	53	21	1	
Waste management	Poor	108	145	2.522 (1.545, 4.119)	0.000*
	Good	62	33	1	

* Significant association as p-value <0.05, COR=crude odds ratio, CI=confidence interval

**Table 5:** multivariate logistic regression analyses of factors associated with diarrheal diseases among under-five children in Hargeisa Internally Displaced Persons Somaliland, 2020

Variable	Categories	Diarrhea occurrence		AOR (95% CI)	P-value
		No	Yes		
Child's age (years)	< 1	51	30	1	
	1 - 2	62	70	3.586 (2.054, 5.196)	0.041*
	3 - 5	57	78	1.143 (0.259, 5.049)	0.860
Exclusive breastfeeding practice	Yes	137	101	1	
	No	33	77	4.006 (3.274, 4.596)	0.018*
Colostrum feeding	Yes	167	111	1	
	No	13	67	36.41 (25.76, 47.9)	0.000*
Water storage container	Jerry can	18	66	4.901 (1.306, 8.387)	0.018*
	Tank	162	112	1	
Hand washing practice of child	Rarely	36	64	5.739 (1.382, 7.822)	0.016*
	Sometimes	60	80	3.201 (1.002, 5.354)	0.042*
	Often	74	34	1	

* Significant association as p-value <0.05, AOR=adjusted odds ratio, CI=confidence interval

Children aged between one and two years were four times more likely to have diarrhea than those aged less than one year (AOR= 3.59, 95% CI: 2.05-5.20). Exclusive breastfeeding practice was also significantly associated with childhood diarrhea. Children who did not receive breast milk were four times more likely to have diarrhea than those who were exclusively breastfed (AOR= 4.01, 95% CI: 3.27-4.60). The risk of developing diarrhea was thirty-six times higher among children who did not receive colostrum milk compared to those who did (AOR= 36.41, 95% CI: 25.76-47.9). Moreover, the children living in households using jerry cans for water storage were 4.9 times more at risk of having diarrhea than those using raised tanks (AOR = 4.90, 95% CI: 1.31-8.39). The risk of developing diarrhea was higher among children who rarely practiced hand washing (AOR= 5.74, 95% CI: 1.38-7.82) and those who sometimes practiced (AOR= 3.20, 95% CI: 1.00, 5.35) compared to children with good handwashing practices.

## Discussion

The two-week prevalence of diarrhea among children under five in Hargeisa IDPs was 51% (95% CI: 46%-56%), an alarming rate significantly higher than the SLHDS 2020 report´s mere 4%. A closely related prevalence of 47% was reported in the Ceel-afwayn district in the Sanag region [11]. In contrast, recent studies in Somalia (11%) [10], Ethiopia (26%) [17], Kenya (19.6%) [16] and Tanzania (12.1%) [18] indicate prevalence rates. This variation may be attributed to several factors, including the focus on urban dwellers; differences in socio-demographic characteristics, environmental factors (such as climate and geographical variations), and behavioral factors (such as the availability and usage of water, latrine, hand washing facilities, and waste disposal practices) specific to the study area.

Children aged between one and two years were approximately four times more likely to experience diarrhea than those aged less than one year (AOR= 3.59, 95% CI: 2.05-5.20), aligning with similar findings in other regions [19-23]. This heightened risk in older children may be attributed to increased morbidity or exposure to infectious agents, coupled with the introduction of complementary feeding, potentially leading to exposure to contaminated food and water.

Exclusive breastfeeding emerged as a significant predictor of reduced diarrheal morbidity in children, providing effective protection against diarrheal disease [24,25]. Children not breastfed exclusively by their mothers were four times more likely to develop diarrhea than their exclusively breastfed counterparts (AOR = 4.01, 95% CI: 3.27-4.60), corroborating findings from other studies [26-29]. This is because of the presence of secretory immunoglobulin A (sIgA) in breast milk. The IgA plays a crucial role in protecting the mucus membranes of the gastrointestinal tract from microbial adherence, highlighting the importance of breastfeeding in preventing childhood diarrhea. There are other protective molecules in breast milk such as IgG, IgM, IgD, lactoferrin, lactoperoxidase, and different kinds of leukocytes.

Colostrum, a sticky white or yellow fluid secreted by the breasts during the second half of pregnancy and early post-birth, provides effective passive immunity to newborns against a wide range of enteric pathogens. They are central to the immunological link that occurs when the mother transfers passive immunity to the offspring [30]. Therefore, this study revealed that the children who did not receive colostrum were thirty-six times more at risk of diarrhea compared to those who were fed colostrum (AOR = 36.41, 95% CI: 25.76-47.9]. While colostrum milk is recognized as a preventive measure for the morbidity of various gastrointestinal infections, its role in treating established infections is not well-documented in the literature [30-34].

The use of unprotected water storage containers poses a potential risk for diarrheal disease transmission. Therefore, the chance of water source contamination depends on whether the container is protected or not. Households using jerry cans for water storage were approximately five times more likely to have children with diarrhea than those utilizing raised tanks (AOR = 4.90, 95% CI: 1.31-8.39). This aligns with a study conducted in Ethiopia [35], but contrasts with another in the same country [36]. This might be because there might be mixed water source usage from both protected and unprotected sources, contamination during transport and storage, or heterogeneity of water sources. Additionally, infrequent hand-washing practices in children emerged as an independent predictor of increased diarrheal morbidity. Children who rarely and sometimes practice hand washing were six times and three times more likely to experience diarrhea, respectively, compared to those who often practice handwashing (AOR = 5.74, 95% CI: 1.38-7.82 and AOR = 3.20, 95% CI: 1.00-5.35). Adequate handwashing plays a crucial role in eliminating microbes from hands, and its neglect increases the risk of exposure through food and drink.

**Limitations:** this study acknowledges certain limitations that should be considered when interpreting the findings. Firstly, the limited availability of literature from surrounding towns specific to the study area posed a challenge. Due to the scarcity of published research on the topic of interest, the study had to rely on existing data, potentially limiting the depth of the literature review. Future research in the region could benefit from a more extensive review of literature, including studies from neighboring towns, to provide a more comprehensive understanding of the context and contributing factors to childhood diarrhea.

Furthermore, the cross-sectional study design employed in this research presents inherent limitations in establishing causal relationships. The nature of cross-sectional studies allows for the assessment of variables at a single point in time, making it challenging to infer cause-and-effect relationships. While associations between certain factors and childhood diarrhea were identified, the study design hinders the ability to determine the temporal sequence of events. To address this limitation, future research could consider longitudinal study designs, allowing for the exploration of causality and the examination of how variables change over time. Longitudinal studies would provide a more robust foundation for understanding the dynamics of childhood diarrhea and the impact of various factors on its occurrence.

## Conclusion

This study sheds light on the alarming prevalence of acute diarrhea among under-five children in Hargeisa IDPs, reaching a striking 51% (95% CI: 46%-56%). The findings reveal a substantial burden of diarrheal diseases, significantly higher than regional and international reports. The identified factors such as lack of exclusive breastfeeding, colostrum discharge, storing drinking water in unprotected containers, and poor handwashing practices underscore the need for targeted interventions. Addressing these determinants through comprehensive health education programs, promoting exclusive breastfeeding, and improving water storage practices can contribute to a substantial reduction in childhood diarrhea.

Based on the study findings, it is recommended that healthcare providers and decision-makers prioritize the promotion of exclusive breastfeeding in the community. Special attention should be given to awareness campaigns emphasizing the importance of colostrum milk and proper handwashing practices. Additionally, interventions to enhance water storage hygiene, such as discouraging the use of unprotected containers like jerry cans, should be implemented. These targeted efforts can contribute to reducing the morbidity of childhood diarrhea in Hargeisa IDPs. Furthermore, future research should explore additional factors contributing to diarrhea, and longitudinal studies could provide insights into the causal relationships and the effectiveness of implemented interventions over time.

### What is known about this topic

- *Diarrheal diseases were endemic and a major concern in Somaliland since 1994;*
- *Personal hygiene and public sanitation are very important to control diarrheal diseases*.

### What this study adds

- *Prevalence of diarrhea was remarkably high (51%) among under-five children in Hargeisa IDPs, offering unique insights specific to this urban population;*
- *Lack of exclusive breastfeeding, colostrum discharge, storing drinking water in unprotected containers, and poor handwashing practices were factors associated with this high prevalence of diarrhea in Hargeisa IDPs*.
